# Establishment and application of a real-time fluorescence quantitative PCR assay for detecting EAPV in *Passiflora edulis*

**DOI:** 10.1371/journal.pone.0356266

**Published:** 2026-09-08

**Authors:** Hanye Zhou, Jie Zhang, Yu Li, Ziran Gao, Muhammad Qasim Aslam, Jin Xu, Yongdui Chen

**Affiliations:** 1 Biotechnology and Genetic Germplasm Resources Research Institute, Yunnan Academy of Agricultural Sciences/Yunnan Provincial Key Laboratory of Agricultural Biotechnology, Kunming, China; 2 Forestry College/Key Laboratory of Forest Disaster Warning and Control in Yunnan Province, Faculty of Biodiversity Conservation, Southwest Forestry University, Kunming, China; 3 School of Ethnic Medicine, Yunnan Minzu University, Kunming, China; 4 Riphah International University, Faisalabad Campus, Faisalabad, Pakistan; Cairo University Faculty of Veterinary Medicine, EGYPT

## Abstract

*East Asian Passiflora virus* (EAPV) is a significant viral pathogen prevalent across passionfruit cultivation regions in China and causes substantial economic losses to the passionfruit industry. The development of a sensitive, rapid, and accurate diagnostic method is essential for virus identification and epidemiological surveillance to support effective disease management strategies. In this study, a SYBR Green-based real-time quantitative PCR (qPCR) assay was developed for EAPV detection using a specific primer set targeting the viral coat protein (CP) gene. The assay was optimized with a primer concentration of 0.2 μmol/L and an annealing temperature of 60°C. The primers exhibited high specificity, generating a standard curve with an amplification efficiency of 90.9% and a coefficient of determination (R^2^) of 0.992. The limit of detection was 11.41 × 10^2^ copies/μL, representing a 1000-fold greater sensitivity than conventional PCR. Moreover, viral accumulation was successfully detected in both inoculated and systemic leaves of passionfruit plants. In 2025, a total of 120 suspected virus-infected passionfruit samples were collected from Yunnan Province, China, and all samples tested positive for EAPV using the developed qPCR assay. Overall, the SYBR Green-based qPCR method established in this study demonstrated high specificity and sensitivity, providing a reliable tool for rapid EAPV diagnosis and epidemiological investigations.

## 1. Introduction

Passionfruit (*Passiflora edulis*), also known as eggfruit, belongs to the genus *Passiflora*, family Passifloraceae, is a perennial, evergreen, woody, climbing vine. It originates from the north-central region of America, and then introduced to China. Currently, it is widely cultivated in tropical and subtropical regions of the country such as Taiwan, Guangxi, Guizhou, Fujian, and Yunnan Province [1,2]. As an economically significant crop, passionfruit is highly valued for its rich nutritional content, distinctive flavor, and notable medicinal properties [3]. However, viral diseases pose a major challenge to the passionfruit industry. In recent years, the extensive introduction and cultivation of passionfruit, coupled with frequent interprovincial and even international trade of seedlings and the continuous expansion of planting areas, has accelerated the spread and transmission of passionfruit viral diseases. This has resulted in extremely severe damage to the passionfruit industry [2]. Particularly, in Guangxi Province, an area infection rates posed by viral diseases have reached as high as 100% [4]. Infected passionfruit plants exhibit symptoms including stunted growth, mottled leaves, yellowing, leaf curling, deformation, and variegation. Fruits become deformed, smaller, and woody, with severe infections potentially halting growth or causing plant death.

Globally, over 40 viruses belonging to 13 genera have been reported to infect passionfruit [3], with the most representative viruses from the genus *Potyvirus* within the family *Potyviridae*. To date, 15 *Potyvirus* species have been reported to infect passionfruit globally [3], with six *Potyvirus* species documented on Chinese passionfruit: *Passionfruit severe mottle-associated virus* (PFSMaV) [5], *Passionfruit mottle virus* (PaMV) [6], *East Asian passiflora virus* (EAPV), *Telosma mosaic virus* (TeMV), *Turnip mosaic virus* (TuMV) [7] and *Passion fruit severe mottle virus* (PFV) [8]. EAPV was first identified on passionfruit in Japan [9] and was first detected in mainland China in 2018 [10]. It is the most commonly reported passionfruit virus in recent years and one of the most significant viruses within the *potyvirus* genus affecting passionfruit, often causing complex infections with other viruses. EAPV is a positive-sense single-stranded RNA virus that causes passionfruit woodiness disease (PWD), characterized by leaf mosaic, fruit malformation, woodiness, and stunted growth [9]. In Japan, EAPV is associated with PWD, which was misidentified as a strain of PWV based on its symptomatology. In Taiwan, the causal agent of PWD was also misidentified as PWV for decades [11] but has been reclassified as EAPV after sorting out the genomic sequences [12].

Researches on the host range of EAPV are limited. Under field conditions this virus has been found associated with passionfruit plants, however under controlled conditions it can readily infect *nicotiana benthamiana* plants [13]. Whether EAPV possesses a broader host range remains unknown. Like other members of *potyvirus* genus such as TeMV, EAPV can be transmitted by insect vectors such as whiteflies and aphids, as well as through various other routes including cuttings, grafting, and mechanical inoculation [14]. As one of the primary pathogens causing woody rot disease in passionfruit, EAPV has become a critical bottleneck constraining the sustainable development of the passionfruit industry in China. Current virus disease control strategies in production primarily involve planting virus-free seedlings and promptly removing infected plants from fields. Therefore, implementing these strategies requires rapid, accurate, and sensitive detection methods as a foundation [15,16], which is crucial for the prevention and control of EAPV.

So far, virus detection technologies primarily include (1) Immunoserological detection, specifically the enzyme-linked immunosorbent assay (ELISA), which is currently the most widely used immunological method in plant virus diagnostics [15]. This method combines the rapid response and convenience of immunofluorescence, making it suitable for large-scale sample testing and plant virus diagnosis. However, it suffers from drawbacks including low specificity and potential false negatives, which may compromise detection accuracy. (2) Molecular biology detection, specifically polymerase chain reaction (PCR) technology [17]. PCR offers high sensitivity, enabling virus detection with minimal sample quantities. Currently, the main molecular detection methods for EAPV in passionfruit include multiplex RT-PCR [18], multiplex RT-LAMP [19] and TC-RT-PCR [20]. The above researches reveal that the sensitivity of different methods for detecting plant viruses varies considerably, with the lowest detection limit being 10 pg/μL total RNA [21]. However, these detection techniques still fail to meet the requirements for the early diagnosis of plant viruses. These approaches may miss latently infected plants, which can serve as reservoirs of inoculum in the field.

Real-time fluorescence quantitative PCR (qPCR), as a highly sensitive, specific, and stable detection technique, has been widely applied in the detection of various plant viruses. This method enables quantitative assessment of viral distribution within plant tissues, holding significant importance for studying viral biological characteristics. qPCR technology has been applied to the detection of TeMV in passionfruit and demonstrates a high degree of sensitivity [22]. This study aims to establish a SYBR Green-based absolute real-time quantitative PCR detection method for EAPV by constructing a standard template from the CP gene region of EAPV and generating standard curves, providing a more sensitive method for the early detection and epidemiological investigation of EAPV. It also provides technical support for the prevention and control of EAPV disease in field-grown passionfruit.

## 2. Materials and methods

### 2.1. Experimental materials

Laboratory-preserved passionfruit samples infected with EAPV alone served as test samples for qPCR primer screening. Previously reported passionfruit-infecting *Potyviruses* from our lab like Potato virus Y (PVY), Telosma mosaic virus (TeMV), and Chilli veinal mottle virus (ChiVMV), were successfully preserved and utilised in this study. Passionfruit samples were collected from the fields of Shizong, Luoping County from Qujing City, as well as Qiubei, Maguan, Malipo and Xichou County from Wenshan City in 2025. A total of 120 passionfruit leaves exhibiting suspected viral disease symptoms such as mottling, wrinkling, and deformation were obtained.

### 2.2. Experimental methods

#### 2.2.1. Total RNA extraction.

Total RNA was extracted from 0.2 g of diseased passionfruit leaves using an RNA extraction kit (Promega, Beijing Biotech, Co., Ltd), following the kit instructions for the specific procedure. The concentration and purity of total RNA were determined using a Thermo Scientific Nanodrop spectrophotometer. The RNA quality was assessed via agarose gel electrophoresis and then stored at −80°C for later use.

#### 2.2.2. Primers design.

Coat protein gene sequences were obtained from the EAPV Yunnan isolate EAPV-FJ (accession number: PV208404) obtained in our group [23]. A pair of specific primers were designed based on conserved regions of the coat protein for RT-qPCR detection of EAPV, qEAPVF (5’-GCTCAGCCAACATTGAGACA-3’) and qEAPVR (5’-GTGCTACCGCTTCTCTTGCT-3’), using the website https://www.bioinformatics.nl/cgi-bin/primer3plus/primer3plus.cgi. The primers were synthesized by Beijing Tsingke Biotech Co., Ltd. After synthesis, the primers were tested for specificity using EAPV-positive samples and passionfruit leaves infected only with PVY, TeMV or ChiVMV respectively.

#### 2.2.3. Preparation of EAPV plasmid standard.

The synthesized cDNA was used as the template for PCR amplification. The 50 μL PCR reaction mixture was prepared containing 25 μL of 2 × ApexHF CL PCR Master Mix (Accurate, Changsha, China), 1 μL each of primers, 5 μL of cDNA template, and volume makeup up to 50 μL by adding DEPC treated water. The PCR reaction was performed under the following conditions: initial denaturation at 94°C for 3 min; followed by 35 cycles of denaturation at 94°C for 30 s, annealing at 50°C for 30 s, and extension at 72°C for 60 s; with a final extension at 72°C for 10 min. The PCR amplicons were separated on a 1% agarose gel, excised, and purified using a gel extraction kit (Takara Bio, Beijing, China) for performing ligation into the pMD^TM^18-T vector. The ligation reaction was transformed into *E. coli* DH5α competent cells by heat shock transformation and subject to bluewhite screening on ampicilline resistance plate. The positive colonies were screened and sent to Sangon Biotech (Shanghai, China) for sequencing. Clone with correct sequencing results was used for plasmid purification and named as EAPV-12. This recombinant plasmid was subsequently used as the standard template for qPCR analysis. Plasmid copy number was calculated as described by Sun et al [24].

#### 2.2.4. Optimization of real-time qPCR detection system.

The qPCR assay was optimized using the recombinant plasmid EAPV-12 as the template. A 20 μL reaction mixture was prepared containing 10 μL of 2 × GoTaq RT-qPCR Master Mix, varying concentrations (0.4, 0.5, 0.6, 0.8, and 1.0 μL) of forward and reverse primers, 1 or 2 μL of plasmid template, and nuclease-free ddH2O added to a final volume of 20 μL. The qPCR amplification conditions consisted of an initial denaturation at 95°C for 10 min, followed by 40 cycles of denaturation at 95°C for 15 s, annealing at six different temperatures (52, 54, 55, 56, 58, and 60°C) for 30 s based on the primer melting temperature (Tm), and extension at 72°C for 30 s. Amplification efficiency and melt curve analyses were performed to determine the optimal primer concentration with template volume and annealing temperature.

#### 2.2.5. Establishment of real-time quantitative PCR standard curve.

For standard curve generation, the EAPV-12 plasmid was used to prepare 10-fold serial dilutions in ddH2O, with concentrations ranging from 11.41 × 10^9^ to 11.41 × 10¹ copies/μL. Nuclease-free water served as the blank control, while virus-free healthy passionfruit samples served as negative controls. Each sample was analyzed in three technical replicates. Following amplification, a standard curve was constructed using the Ct values obtained from amplification and the logarithmic values of the corresponding plasmid copy numbers [25].

#### 2.2.6. Sensitivity and primer specificity testing of the qPCR detection system.

Sensitivity of conventional PCR and RT-qPCR was compared, using ten-fold serial dilutions of plasmid standards as templates, starting from an initial concentration of 11.41 × 10^9^ copies/μL. For specificity testing of the EAPV qPCR assay, cDNA samples confirmed to contain PVY, TeMV, and ChiVMV but not EAPV were employed.

#### 2.2.7. Detection of virus accumulation in passionfruit leaves.

EAPV accumulation and systemic movement in infected passionfruit plants was assessed by measuring viral load in inoculated and systemic leaves at different time points using qPCR assay. Initially, four-leaf-stage seedlings of passionfruit that were first confirmed free of EAPV via RT-qPCR were sap inoculated using leaf extract of EAPV infected passionfruit plant and two leaves from each plant were inoculated at the same position. For qPCR assay, leaf samples were collected in triplicate from inoculated and systemic leaves at 0, 3, 7, 14, 21 and 28 days post-inoculation (dpi). Uninoculated virus-free passionfruit leaves collected at 0 dpi served as the normal control (CK).

For inoculated leaves and systemic leaves at different time points following viral inoculation, three biological replicates were selected, with three technical replicates set up for each biological replicate; the mean of the technical replicates was then took as the value for each biological replicate. Both the Ct values and the calculated copy numbers represent the mean of the values from the three biological replicates. These values were imported into Prism 10.1.2 software for analysis.

#### 2.2.8. Field sample testing.

Following the establishment of the qPCR assay, comparative field sample testing was conducted alongside conventional PCR detection. A total of 120 passionfruit samples suspected of EAPV infection were collected from different counties in Qujing and Wenshan, China, for assay evaluation.

## 3. Results

### 3.1. Specificity analysis of EAPV primers

**Fig 1:**
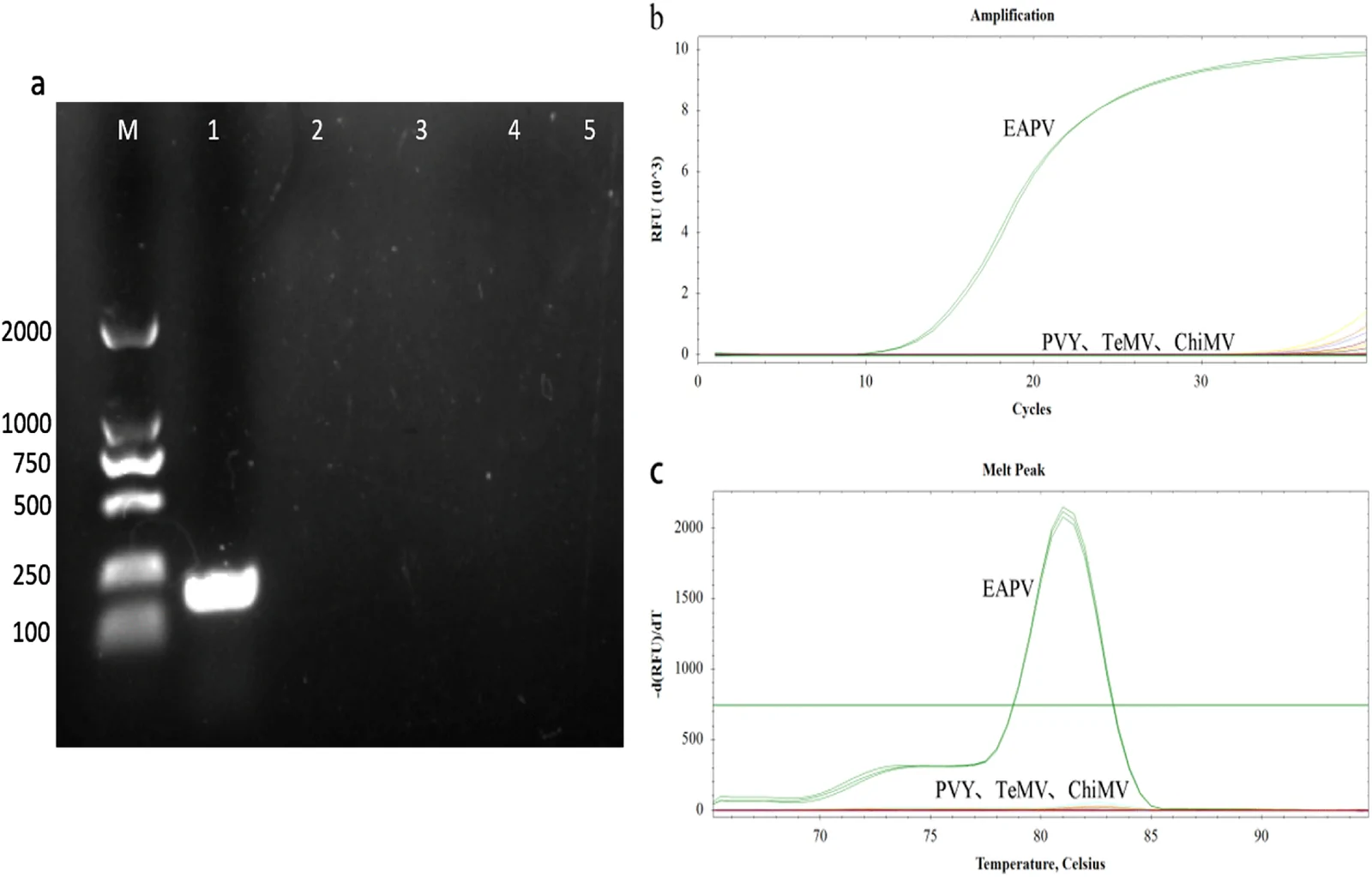
Specific detection of EAPV using qPCR and conventional PCR assay. (a) Lane 1 represents an EAPV positive sample, lane 2-5 indicates PVY, TeMV, ChiVMV, and healthy passionfruit leaves; (b) Amplification curves obtained from real-time qPCR assay using EAPV-infected samples and samples infected with other viruses. A strong amplification signal was observed only in the EAPV-positive sample. (c) Melting peak analysis showed a single distinct peak for the EAPV-positive sample at approximately 81–82 °C, indicating specific amplification of the target product.

Samples that were asymptomatic and negative according to PCR tests for PVY, TeMV, and ChiVMV were regarded as healthy passionfruit samples. RT-PCR results showed that EAPV-infected samples produced a band of approximately 200 bp, while negative controls and samples infected with PVY, TeMV, or ChiVMV exhibited no bands, suggesting the specificity of EAPV-CP specific primers (Fig 1a). Cloning and sequencing of the target band revealed high sequence identity with the EAPV CP gene, confirming the specificity of primers qEAPVF/qEAPVR. In qPCR assay, passionfruit samples carrying PVY, TeMV, ChiVMV, or EAPV, only the EAPV-carrying sample exhibited a normal amplification curve, while other viral samples showed no significant amplification curve (Fig 1b), further emphasizing the specificity particular primer pairs. Melt curve analysis revealed that all peaks produce melting at 81.5°C (Fig 1c). These results confirm that the primer pair qEAPVF/qEAPVR can be used for the specific detection of EAPV in conventional and qPCR assay.

### 3.2. Establishment of the standard curve of SYBR green-based qPCR system

Using the EAPV plasmid standard as a template, the primer concentration, template amount, and annealing temperature were optimized for qPCR assay. The optimal reaction conditions were determined to be 0.2 μmol/L for both forward and reverse primers and an annealing temperature of 60°C, which produced stable amplification curves and a single specific melting peak. The optimized 20 μL reaction mixture consisted of 10 μL 2 × GoTaq RT-qPCR Master Mix (Promega, Beijing, China), 0.4 μL each of forward and reverse primers, 2 μL plasmid template, and ddH2O added to a final volume of 20 μL. The reaction profile included an initial denaturation at 95°C for 10 min, followed by 40 cycles of 95°C for 15 s, 60°C for 30 s, and 72°C for 30 s.

**Fig 2:**
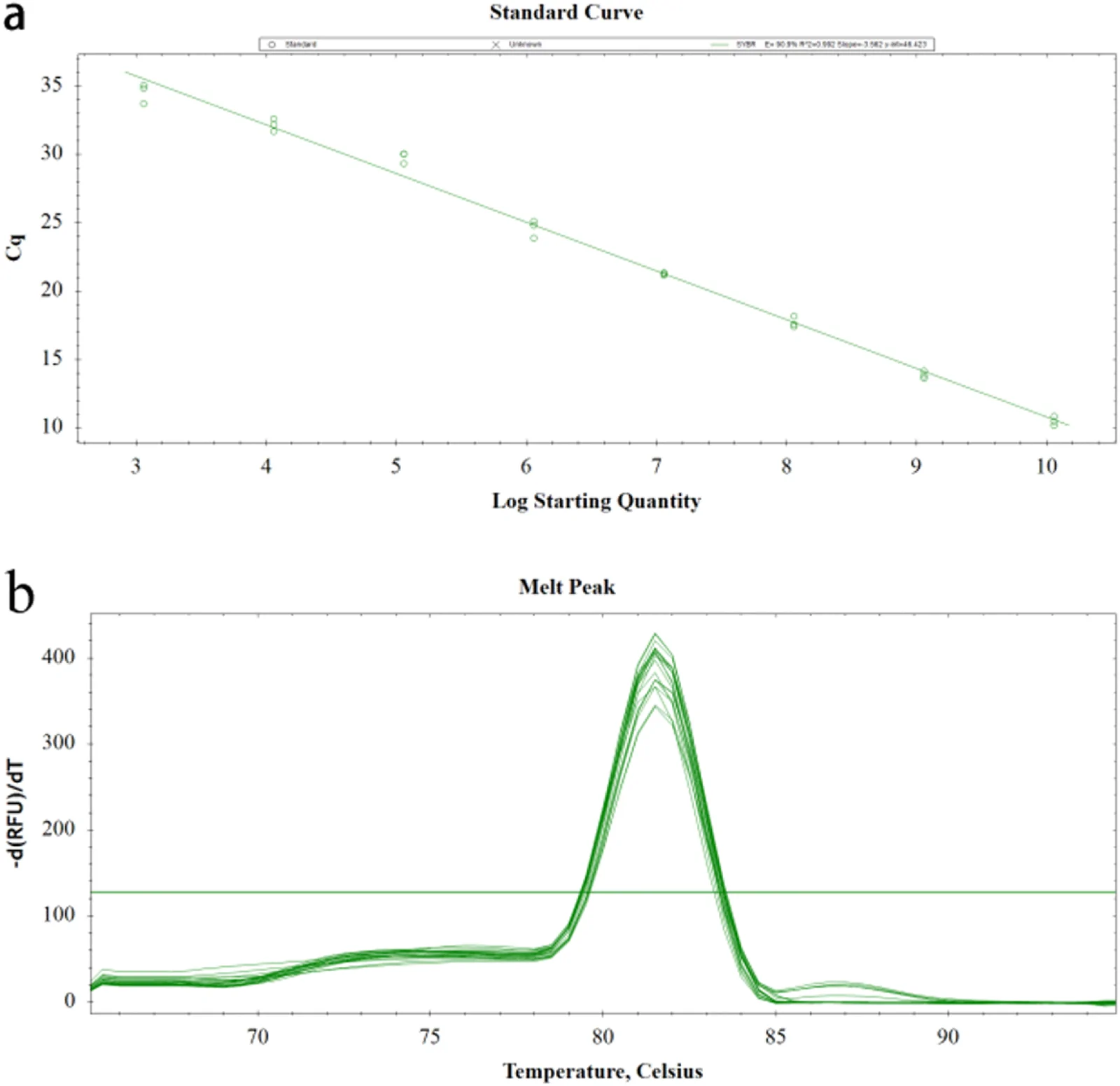
Establishment of qPCR standard curve for the EAPV (a) Standard curve: The X-axis represents the copies number, which ranges from 11.41 × 10^2^ copies/pl. to 11.41 × 10^9^ copies/pl. The corresponding Ct values are represented on the Y-axis. (b) Melting curve analysis of the qPCR products showing a single specific melting peak at approximately 81 ± 0.5°C.

Using the optimized qPCR system, plasmid standards ranging from 11.41 × 10^2^ to 11.41 × 10^9^ copies/μL showed a strong linear relationship between Ct value and template concentration. The standard curve exhibited a slope of −3.562, a correlation coefficient (R^2^) of 0.992, and an amplification efficiency of 90.9%. The standard curve equation was y = −3.562x + 42.834, where y represents the Ct value and x represents the logarithm of the plasmid copy number (Fig 2a). Melting curve analysis showed a single specific peak with a Tm of 81 ± 0.5°C for all positive samples, indicating the absence of non-specific amplification and primer-dimer formation (Fig 2b).

### 3.3 Comparison of sensitivity between qPCR and conventional PCR for EAPV detection

**Fig 3:**
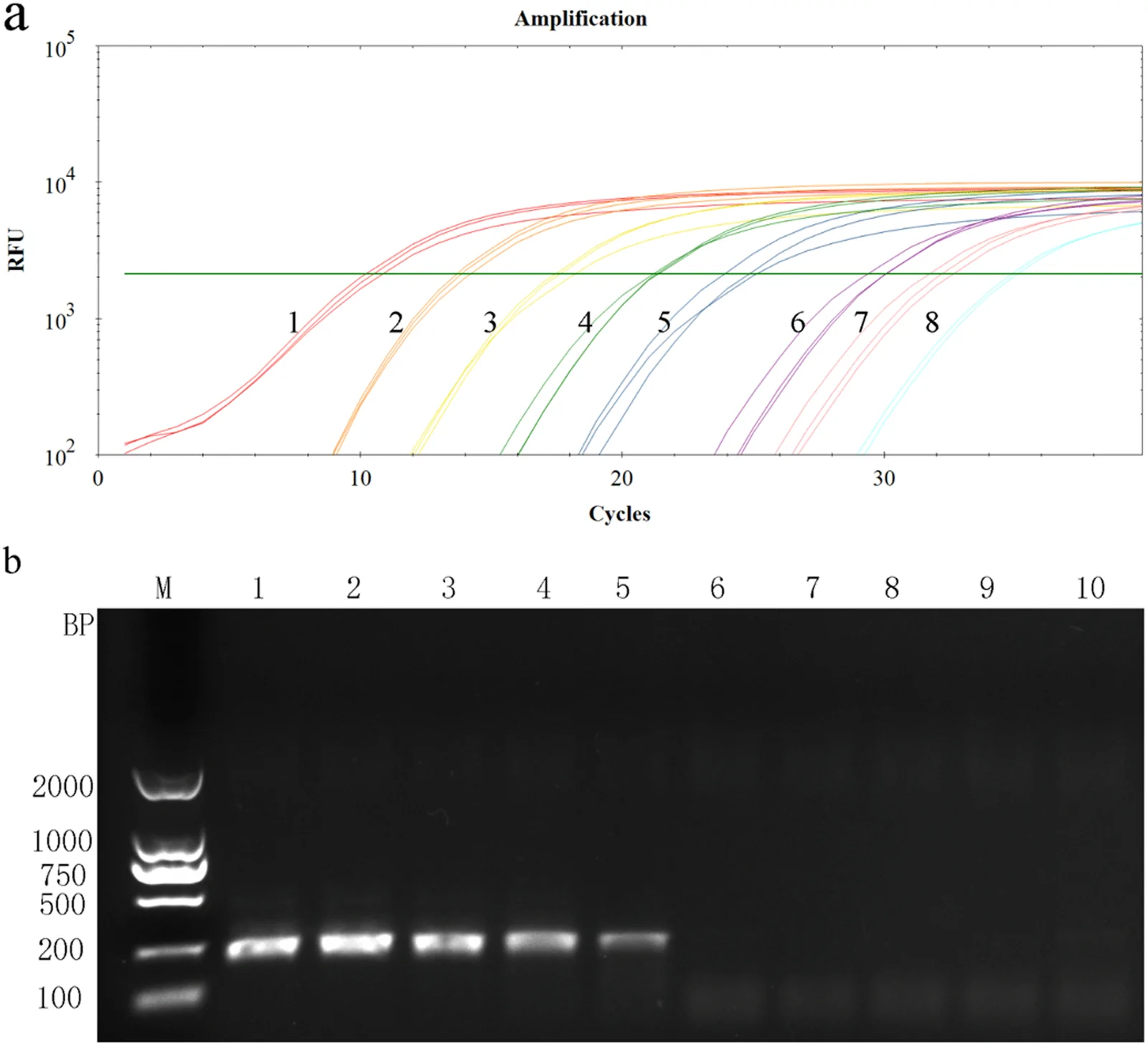
Comparison of sensitivity between real-time qPCR and conventional PCR. (a) Amplification curves obtained from the RT-qPCR assay using 10-fold serial dilutions of the EAPV plasmid standard. A plot of amplification: X-axis represents the cycle threshold (Ct) values, the Y-axis represents the fluorescence intensity. Curves 1–8 correspond to 10-fold serial dilutions of EAPV plasmid standard, ranging from 11.41 × 10^9^ to 11.41 × 10^2^ copies/μL, respectively. (b) Amplification results of conventional PCR. M is 2000 bp DNA Maker; Lane 1-9 corresponds to 10-fold serial dilutions of EAPV plasmid, where lane 5 depicted the PCR amplification with minimum standard concentration (11.41 × 10^5^ copies/μL), lane 10 is ddH_2_O containing negative control with no amplification.

An EAPV plasmid standard with an initial concentration of 11.41 × 10^9^ copies/μL was serially diluted tenfold. Each dilution was used as a template for both conventional PCR and qPCR amplification. Results showed that qPCR could detect EAPV plasmid standard at a concentration of 11.41 × 10^2^ copies/μL (Fig 3a), while conventional PCR could detect it at a minimum concentration of 11.41 × 10^5^ copies/μL (Fig 3b). The results indicate that our optimized qPCR detection system was 1000 times more sensitive than that of conventional PCR (Table 1).

**Table 1:** Comparison between qPCR and conventional PCR results for the detection of EAPV in standard plasmids.

Standard Plasmid/Samples		Conventional PCR	qPCR/ (copies/μL)
Standard Plasmid	11.41 × 10^9^	+	+
	11.41 × 10^8^	+	+
	11.41 × 10^7^	+	+
	11.41 × 10^6^	+	+
	11.41 × 10^5^	+	+
	11.41 × 10^4^	−	+
	11.41 × 10^3^	−	+
	11.41 × 10^2^	−	+
Sample	PVY	−	−
	TeMV	−	−
	ChiVMV	−	−
	Healthy passion fruit leaves	−	−

### 3.4. Detection of EAPV accumulation in passionfruit leaves

**Fig 4:**
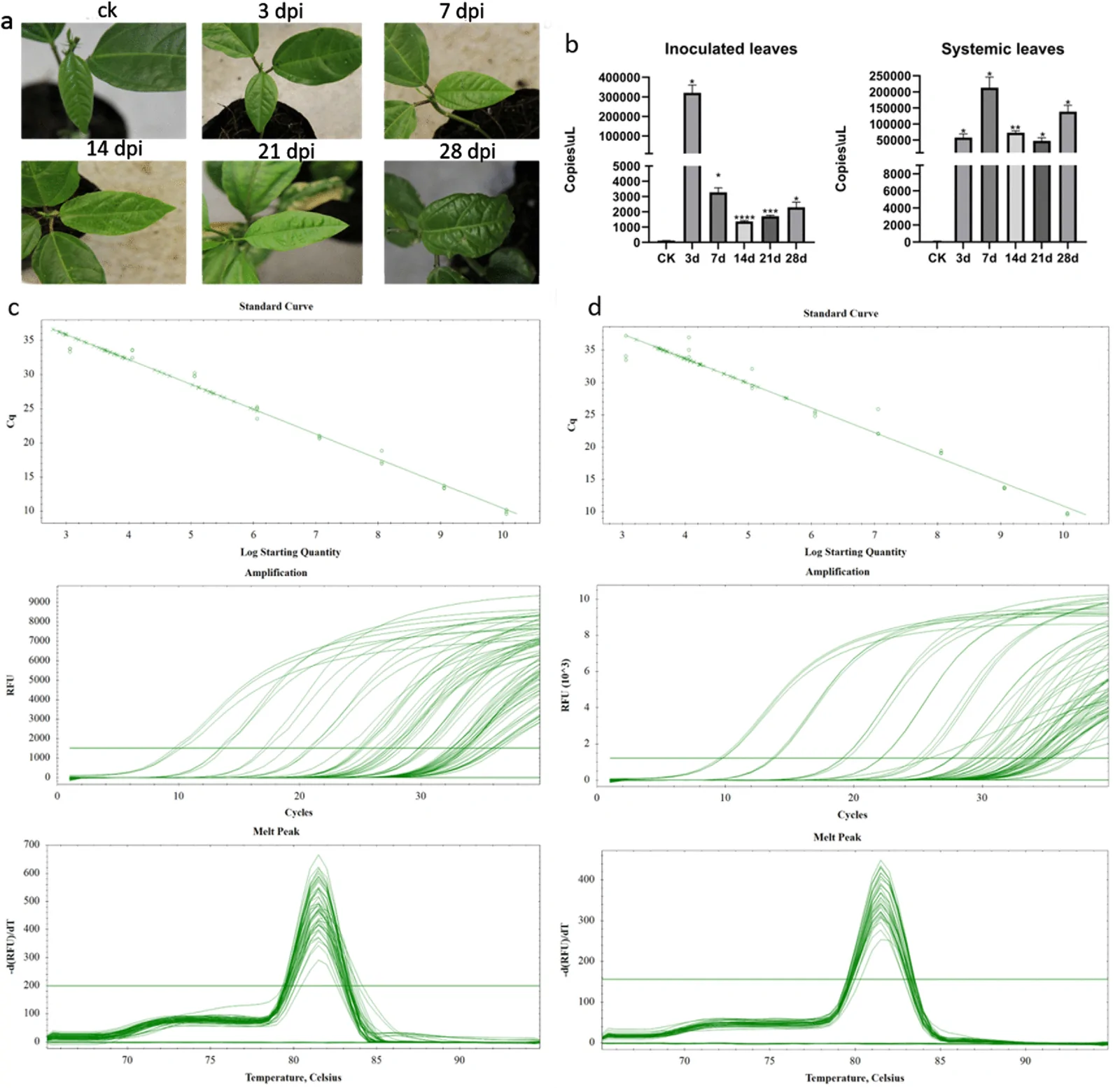
EAPV detection of passionfruit leaves. (a) Symptoms of passionfruit systemic leaves after EAPV inoculation at 0, 3, 7, 14, and 28 dpi. (b) The accumulation of EAPV in inoculated and systemic leaves of passionfruit at 0, 3, 7, 14, 21 and 28 dpi. qPCR specific standard curve, amplification curve, and melt curve for EAPV in passionfruit (c) inoculated leaves and (d) systemic leaves. Differences in viral copy numbers between inoculated and systemic leaves at different time points following viral inoculation were analyzed using ANOVA and Tukey’s HSD test for multiple comparisons. An α-level of <0.05 was used as the significance threshold in all analyses, and all values are reported as Mean ± SD. * indicates P < 0.05, ** indicates P < 0.01, **** indicates P < 0.0001.

The accumulation of EAPV in inoculated and systemic leaves at 3, 7, 14, 21, and 28 dpi was quantified using the established RT-qPCR assay. For this purpose, leaves of passionfruit plants were sap-inoculated with EAPV inoculum. Following EAPV inoculation, systemic leaves of passionfruit plants began to exhibit vein chlorosis at 14 days post-inoculation (dpi). At 21 dpi, chlorosis and leaf curling symptoms became apparent, and the severity of leaf curling further increased by 28 dpi. Despite symptom development, the infected plants remained viable throughout the observation period (Fig 4a).

In inoculated leaves, the mean Ct values at 3, 7, 14, 21, and 28 dpi were 26.49, 33.99, 35.28, 34.93, and 34.47, respectively, corresponding to viral accumulation levels of 3.4 × 10^5^, 3.1 × 10^3^, 1.4 × 10^3^, 1.7 × 10^3^, and 2.3 × 10^3^ copies/μL (Fig 4b). These results suggested that viral accumulation in inoculated leaves was highest at 3 dpi and declined markedly after 7 dpi with a slight increase observed at 21dpi. In systemic leaves, the average Ct values at 3, 7, 14, 21, and 28 dpi were 30.82, 28.66, 30.44, 31.17, and 29.38, respectively, corresponding to EAPV accumulation levels of 5.8 × 10^4^, 2.1 × 10^5^, 7.3 × 10^4^, 4.7 × 10^4^, and 1.4 × 10^5^ copies/μL (Fig 4b). Viral accumulation in systemic leaves reached its highest level at 7 dpi, gradually decreased thereafter, and slightly increased again at 28 dpi. In both systemic and inoculated leaves, the negative control (CK) produced only late amplification signals at 81 ± 0.5°C with the Ct value of 39.30 (Fig 4c & 4d), which were considered background amplification and were excluded from positive detection.

### 3.5. Detection of EAPV in field passionfruit samples

**Fig 5:**
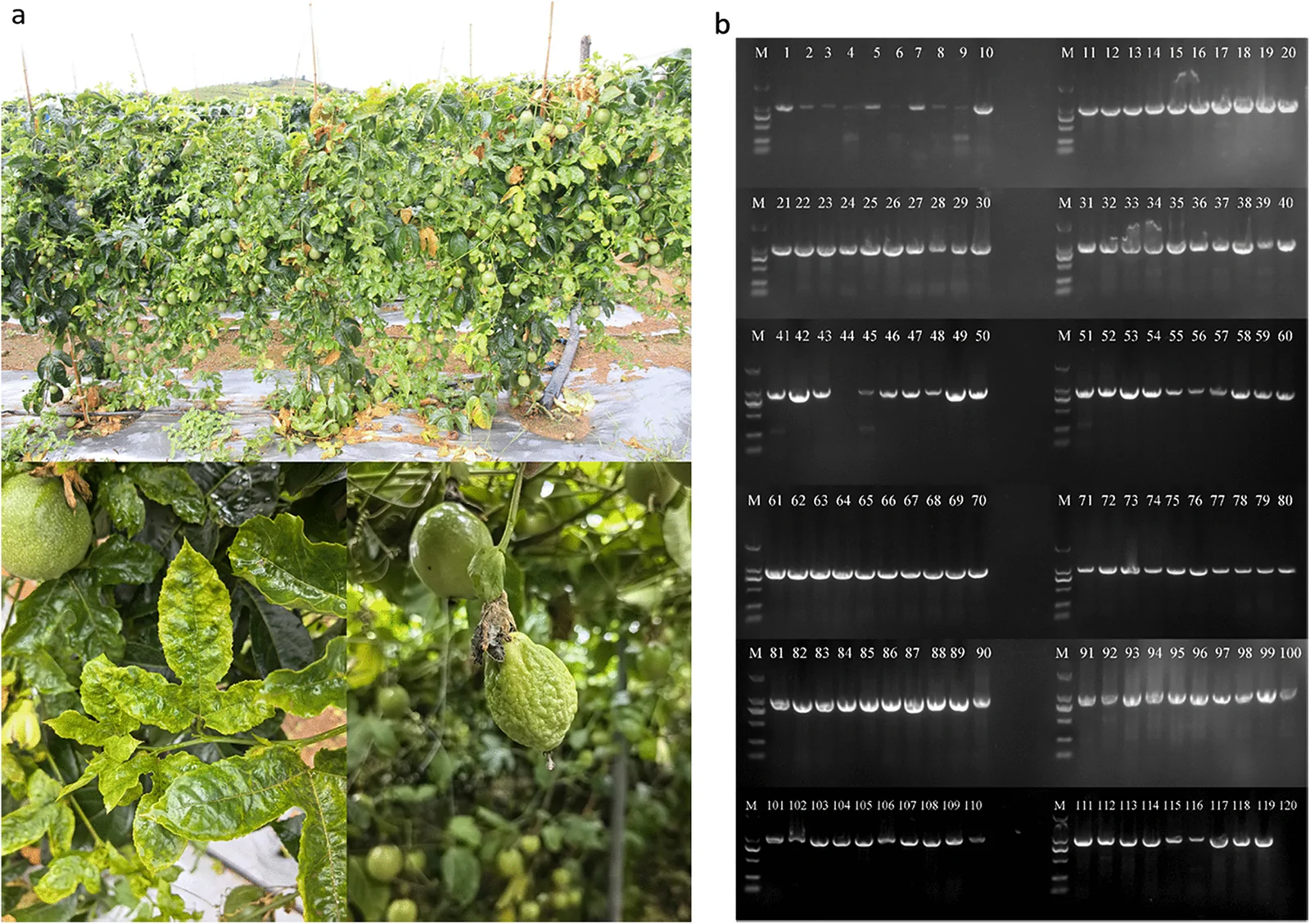
Symptoms of passionfruits infected with viral diseases in the fields and RT-PCR amplification electrophoresis of EAPV. (a) Passionfruit viral disease caused severe damage in the fields. Infected leaves exhibit symptoms such as mosaic, chlorosis and wrinkling, while the fruits show malformation and woodiness. (b) The agarose gel indicates that the RT-PCR amplification products correspond to the expected target gene size. M is 2000 bp DNA Maker; Samples 1-40 are collected from Qiubei County, samples 41-70 are collected from Maguan County, samples 71-80 are collected from Malipo County, samples 81-100 are collected from Shizong County, samples 101-110 collected from Luoping County, and samples 111-120 are collected from Xichou County, respectively.

A total of 120 passionfruit leaf samples exhibiting typical virus-like symptoms (Fig 5a) were collected and analyzed using both conventional RT-PCR and the established qPCR assay. Following amplification and analysis by 1% agarose gel electrophoresis, PCR results showed that 117 of the 120 samples produced the expected target band corresponding to EAPV, whereas no amplification was detected in the remaining three samples (Fig 5b). In contrast, the qPCR assay successfully detected EAPV in all 120 field samples, resulting in a detection rate of 100%, compared with 97.5% obtained by conventional RT-PCR (Table 2). These results demonstrate that the developed qPCR assay possesses higher sensitivity and detection accuracy than conventional PCR for the detection of EAPV in field-collected passionfruit samples.

**Table 2:** Comparison of EAPV detection results by conventional PCR and qPCR in passionfruit samples from the fields.

Region	Sample Numbers	Number of positive samples		Detection rate/%	
		RT-PCR	RT-qPCR	RT-PCR	RT-qPCR
Qiubei County	40	39	40	97.5	100
Maguan County	30	29	30	96.7	100
Malipo County	10	10	10	100	100
Shizong County	20	20	20	100	100
Luopin County	10	10	10	100	100
Xichou County	10	9	10	90	100
Total	120	117	120	97.5	100

## 4. Discussion

In recent years, EAPV has been frequently reported in passionfruit-growing regions of China. Infection with EAPV leads to symptoms such as mottled and wrinkled leaves, fruit deformities, and, in severe cases, plant death. These effects pose a significant threat to the passionfruit industry. In China, EAPV is widely distributed across passionfruit growing regions. As the predominant virus infecting passionfruit in Guangxi, its detection rate reached 73.76% by DAS-ELISA and RT-PCR method [4]. In Fujian, EAPV has become one of the three major viral pathogens, detected in 21.67% of 60 suspected diseased samples when employing TC-RT-PCR approach [20]. In Zhanjiang, Guangdong, field surveys identified EAPV as a major virus detected in passionfruit crops with conventional PCR technology [26]. This study establishes, for the first time, a real-time fluorescent quantitative PCR detection method for rapid, highly sensitive detection of EAPV in field passionfruit samples.

RT-qPCR is currently the core technology for plant virus detection, offering both high sensitivity and specificity, and providing significant advantages over other traditional or emerging detection methods. Conventional RT-PCR and multiplex RT-PCR have minimum detection limits of approximately 10 pg/μL of total RNA, which only meets the qualitative detection requirements for EAPV [18,20]; Dot-ELISA and Tissue print-ELISA are susceptible to false positives due to antibody specificity and sample impurities [27]; RT-LAMP has a detection limit of approximately 145 × 10 ^⁻ 3^ ng/μL total RNA, showing sensitivity comparable to conventional RT-PCR but with limited quantitative capability and insufficient result stability [19]; Although TC-RT-PCR exhibits high sensitivity, it only enables qualitative detection and involves cumbersome procedures, making it unsuitable for research scenarios such as dynamic monitoring of viral infection [20]. Conventional PCR remains the most widely used method for detecting EAPV, but it faces limitations when detecting low viral concentrations in plant tissues. The primers used in this study exhibit high specificity, avoiding cross-reaction with other viruses within the same genus, enabling specific detection of EAPV. However, it should be noted that the specificity conclusions for EAPV may be somewhat limited, as the study did not include testing against all potyviruses infecting passionfruit.

A standard curve with a coefficient of determination (R^2^) of 0.992 was established over a concentration range of 11.41 × 10^9^ copies/μL to 11.41 × 10^2^ copies/μL. In terms of sensitivity, the SYBR Green-based RT-qPCR method established in this study can specifically detect EAPV with a sensitivity 1000-fold higher than that of conventional PCR. These findings are consistent with that of Gu et al., who reported that RT-qPCR was 1000 times sensitive than conventional PCR for detecting TeMV in passionfruit [22]. Throughout the SYBR Green-based RT-qPCR process, we ensured consistency in sample quality (0.2 g of tissue for RNA extraction), total RNA input (1000 ng for reverse transcription into cDNA), cDNA dilution factor, and detection sample volume. These measures minimized variability and ensured the accuracy of the final quantitative results. RT-qPCR technology offers high sensitivity and precise quantification of viral expression in infected samples. Its strong specificity ensures accurate detection without false positives, making it suitable for large-scale sample testing. Moreover, RT-qPCR has irreplaceable applications: it not only enables rapid detection and viral quantification of EAPV but also supports advanced research such as screening for disease-resistant variety, evaluating pesticide efficacy, and investigating molecular mechanisms of viral infection.

In this study, following EAPV inoculation, viral accumulation in inoculated leaves was highest at 3 dpi and subsequently declined gradually. This early high viral load may be associated with residual inoculum remaining on the mechanically inoculated leaves. Thereafter, activation of host defense responses may have contributed to the reduction in viral accumulation until 21 dpi, after which viral content increased slightly again. Concurrently, disease symptoms on the inoculated leaves progressively intensified, with noticeable leaf curling and mosaic symptoms developing during the later stages of infection. In systemic leaves, EAPV accumulation reached its highest level at 7 dpi, although no obvious symptoms were observed at this stage. Viral accumulation gradually decreased after 7 dpi; however, vein chlorosis became visible from 14 dpi, followed by localized leaf wrinkling at 21 dpi. By 28 dpi, more severe wrinkling of the entire leaf was observed, accompanied by a renewed increase in viral accumulation in systemic tissues. The dynamic changes in viral accumulation observed in systemic leaves may be associated with differences in symptom development and the interaction between viral replication and host defense responses during disease progression [28]. The accumulation pattern of EAPV in inoculated leaves and systemic leaves—initially increasing, then decreasing, and subsequently rising again—differs from the findings of Gu et al [22]. They observed that TeMV, which also belongs to the genus *potyvirus*, exhibited a general upward trend in systemic accumulation following inoculation into passionfruit leaves. The differences in accumulation patterns observed in leaves post-inoculation suggest potential influences of viral biological characteristics and host defense mechanisms. However, the infection mechanism of EAPV in passionfruit remains unclear, and the host resistance responses to viral infection require further investigation.

Using the qPCR method, we tested 120 field samples suspected of viral infection collected from different passionfruit growing areas in Yunnan Province, China in September 2025. Conventional PCR detected EAPV infection in 117 samples, whereas qPCR detected EAPV in all samples. This indicates that qPCR is more sensitive than conventional PCR, and can detect samples with lower virus accumulation. Furthermore, our findings confirm that EAPV is one of the primary viruses infecting passionfruit, consistent with previous studies [2,3,7].

## 5. Conclusion

In conclusion, we have for the first time successfully established and applied a SYBR Green-based qPCR system for molecular detection of EAPV. This novel detection method will facilitate future epidemiological studies and meet the requirement for “early detection and early intervention” of viral diseases. It holds practical value for the prevention and control of the EAPV in the fields.

## Supporting information

S1 Fig Raw images. (PDF)

S1 Data qPCR original data. (XLSX)
