# Supplementary material for: Establishment and application of a real-time fluorescence quantitative PCR assay for detecting EAPV in Passiflora edulis
Source: PLoS One. 2026 Sep 8;21(9):e0356266. doi: 10.1371/journal.pone.0356266 (PMC13552778; doi:10.1371/journal.pone.0356266)
Supplement: Supplementary file 1 [file pone.0356266.s001.pdf]

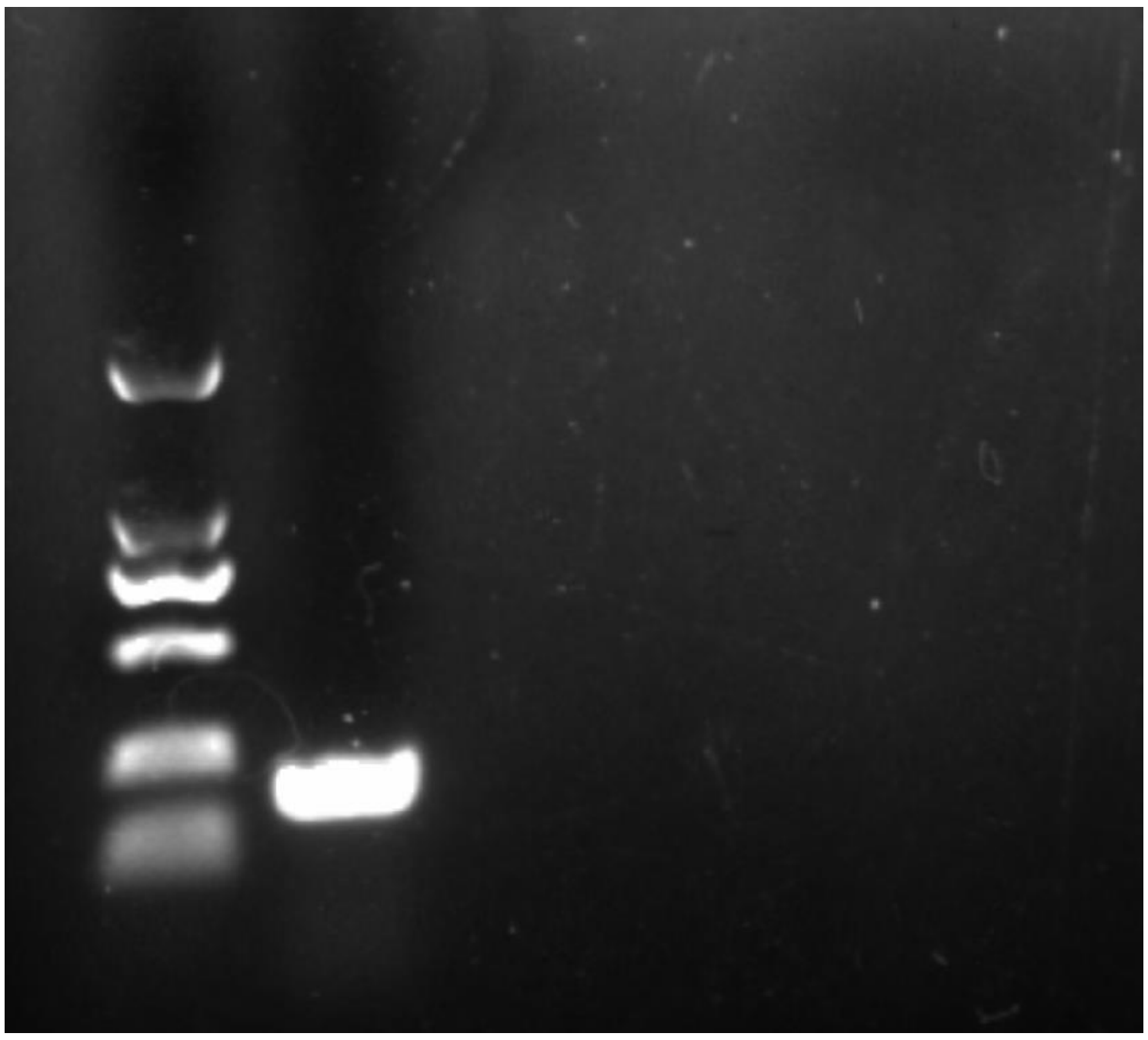

The specificity of EAPV primers tested by conventional PCR

This image generated Fig 1a; Loading order: Marker, EAPV, PVY, TeMV, ChiVMV, healthy; Identity of experimental samples: Different viruses and healthy sample; Method used to capture the image: Bio-Rad Gel Doc XR^+ with image lab^+ Software; Molecular weights of marker are 2000, 1000, 750, 500, 250 and 100 bp, respectively.

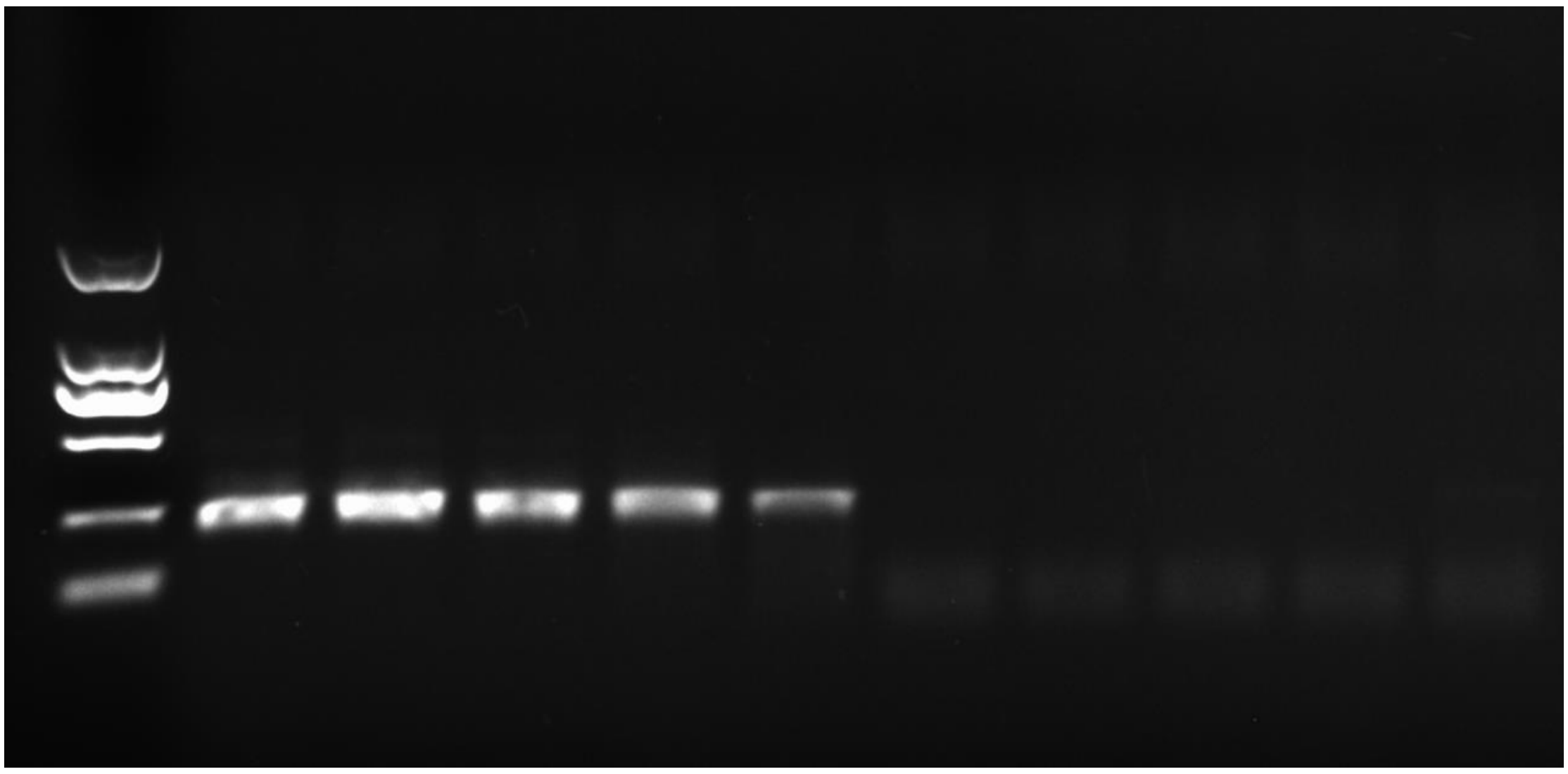

Conventional PCR amplification of EAPV plasmids at different concentrations

Loading order: Marker, lane 1-9 corresponds to 10-fold serial dilutions of EAPV plasmid; Identity of experimental samples: Different concentration of EAPV plamids and ddH_2O; Method used to capture the image: Bio-Rad Gel Doc XR^+ with image lab^+ Software; This image generated Fig 3b; Molecular weights are 2000, 1000, 750, 500, 250 and 100 bp, respectively.

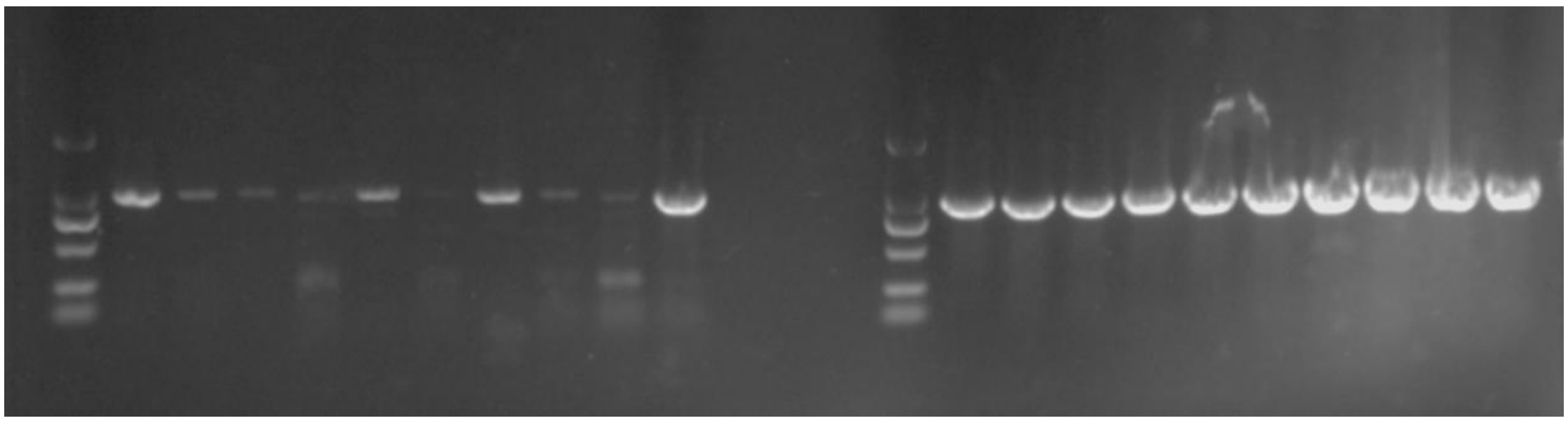

Passionfruit samples 1–20 suspected of being infected with EAPV Loading order: Marker, samples 1-10, marker, samples 11-20; Identity of experimental samples: Different samples; Method used to capture the image: Bio-Rad Gel Doc XR^{+} with image lab^{+} Software; This image generated Fig 5b; Molecular weights are 2000, 1000, 750, 500, 250 and 100 bp, respectively.

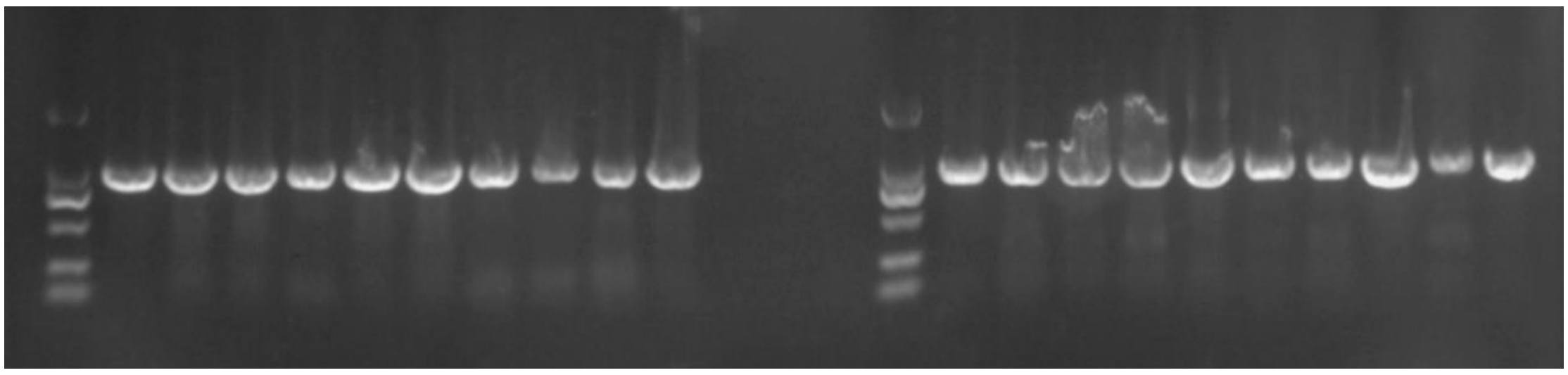

Passionfruit samples 21–40 suspected of being infected with EAPV

Loading order: Marker, samples 21-30, marker, samples 31-40; Identity of experimental samples: Different samples; Method used to capture the image: Bio-Rad Gel Doc XR^{+} with image lab^{+} Software; This image generated Fig 5b; Molecular weights are 2000, 1000, 750, 500, 250 and 100 bp, respectively.

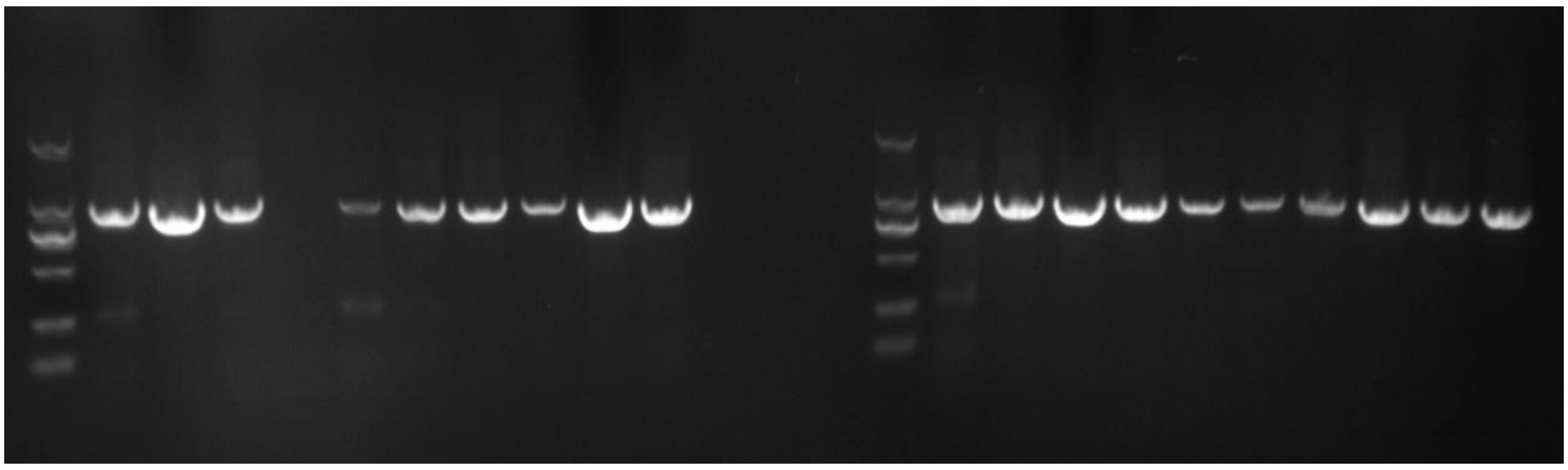

Passionfruit samples 41–60 suspected of being infected with EAPV

Loading order: Marker, samples 41-50, marker, samples 51-60; Identity of experimental samples: Different samples; Method used to capture the image: Bio-Rad Gel Doc XR^{+} with image lab^{+} Software; This image generated Fig 5b; Molecular weights are 2000, 1000, 750, 500, 250 and 100 bp, respectively.

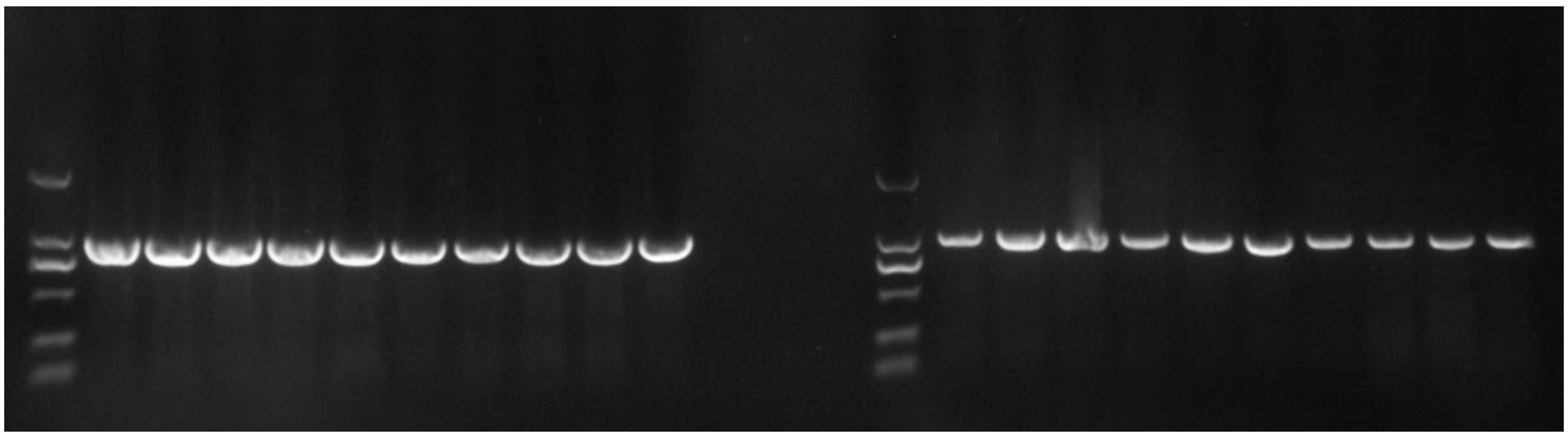

Passionfruit samples 61–80 suspected of being infected with EAPV

Loading order: Marker, samples 61-70, marker, samples 71-80; Identity of experimental samples: Different samples; Method used to capture the image: Bio-Rad Gel Doc XR$^+$ with image lab$^+$ Software; This image generated Fig 5b; Molecular weights are 2000, 1000, 750, 500, 250 and 100 bp, respectively.

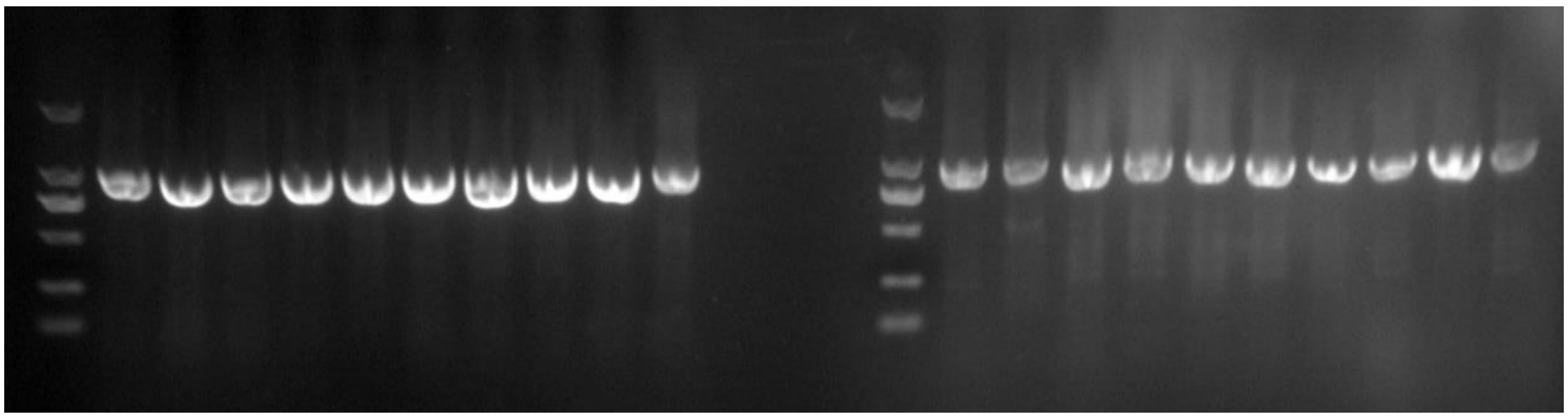

Passionfruit samples 81–100 suspected of being infected with EAPV

Loading order: Marker, samples 81-90, marker, samples 91-100; Identity of experimental samples: Different samples; Method used to capture the image: Bio-Rad Gel Doc XR^+ with image lab^+ Software; This image generated Fig 5b; Molecular weights are 2000, 1000, 750, 500, 250 and 100 bp, respectively.

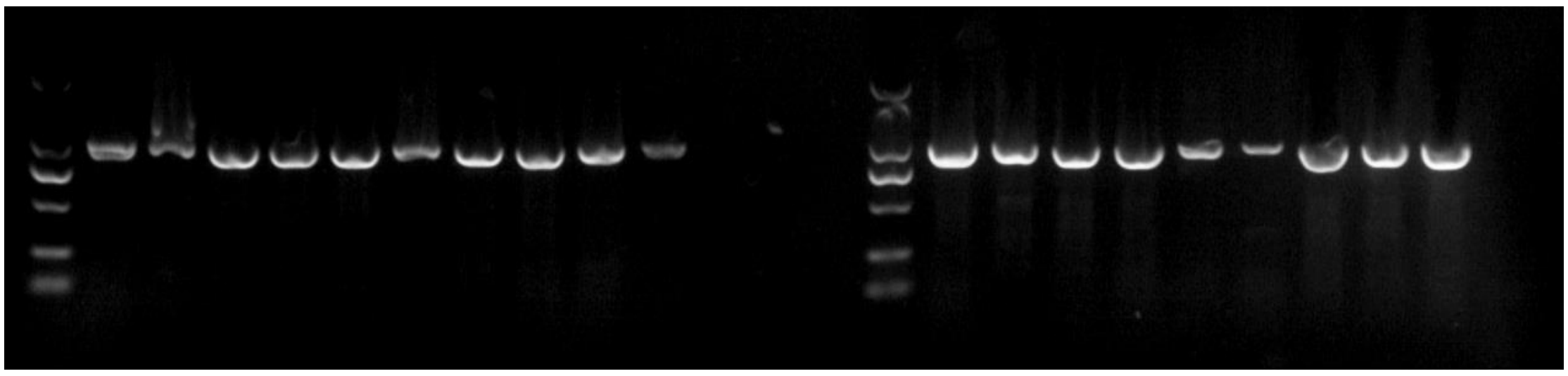

Passionfruit samples 101–120 suspected of being infected with EAPV

Loading order: Marker, samples 101-110, marker, samples 111-120; Identity of experimental samples: Different samples; Method used to capture the image: Bio-Rad Gel Doc XR^{+} with image lab^{+} Software; This image generated Fig 5b; Molecular weights are 2000, 1000, 750, 500, 250 and 100 bp, respectively.
